# Presence and utility of IgA-class antibodies to cyclic citrullinated peptides in early rheumatoid arthritis: the Swedish TIRA project

**DOI:** 10.1186/ar2449

**Published:** 2008-07-04

**Authors:** Anna Svärd, Alf Kastbom, Åsa Reckner-Olsson, Thomas Skogh

**Affiliations:** 1 Rheumatology Clinic, Falu Hospital, SE-791 82 Falun, Sweden; 2 AIR/Rheumatology Unit, Department of Clinical and Experimental Medicne, Faculty of Health Sciences, Linköping University Hospital, SE-581 85 Linköping, Sweden

## Abstract

**Introduction:**

The present study was carried out to assess whether IgA-class antibodies against cyclic citrullinated peptides (IgA anti-CCP) in recent-onset rheumatoid arthritis add diagnostic and/or prognostic information to IgG anti-CCP analysis.

**Methods:**

Serum samples were obtained from 228 patients with recent-onset (<12 months) rheumatoid arthritis at the time of inclusion in the Swedish TIRA cohort (Swedish Early Intervention in Rheumatoid Arthritis). Sera from 72 of these patients were also available at the 3-year follow-up. Disease activity and functional ability measures (erythrocyte sedimentation rate, serum C-reactive protein, 28-joint count Disease Activity Score, physician's assessment of disease activity, and the Swedish version of the Health Assessment Questionnaire) were registered at inclusion and at regular follow-ups during 3 years. An IgA anti-CCP assay was developed based on the commercially available IgG-specific enzyme immunoassay from EuroDiagnostica (Arnhem, the Netherlands), replacing the detection antibody by an anti-human-IgA antibody. A positive IgA anti-CCP test was defined by the 99th percentile among healthy blood donors.

**Results:**

At baseline, a positive IgA anti-CCP test was observed in 29% of the patient sera, all of which also tested positive for IgG anti-CCP at a higher average level than sera containing IgG anti-CCP alone. The IgA anti-CCP-positive patients had significantly higher disease activity over time compared with the IgA anti-CCP-negative patients. After considering the IgG anti-CCP level, the disease activity also tended to be higher in the IgA anti-CCP-positive cases – although this difference did not reach statistical significance. The proportion of IgA anti-CCP-positive patients was significantly larger among smokers than among nonsmokers.

**Conclusion:**

Anti-CCP antibodies of the IgA class were found in about one-third of patients with recent-onset rheumatoid arthritis, all of whom also had IgG anti-CCP. The occurrence of IgA-class antibodies was associated with smoking, and IgA anti-CCP-positive patients had a more severe disease course over 3 years compared with IgA anti-CCP-negative cases. Although IgA anti-CCP analysis does not seem to offer any diagnostic information in addition to IgG anti-CCP analysis, further efforts are justified to investigate the prognostic implications.

## Introduction

Rheumatoid arthritis (RA) is a chronic disabling inflammatory disease with increased risk of premature death, mainly due to coronary vascular disease [1], but modern therapeutic strategies in early RA have improved the prognosis considerably [2-4]. Since, however, the clinical manifestations and consequences of RA vary between individuals, as do the responses to therapy, there is an urge to obtain reliable predictors of disease course/outcome and therapy response, in order to allow rational individually tailored therapy regimens. Furthermore, the high direct costs to society for biological agents place further emphasis on the need for reliable predictors.

The discovery of anti-citrullinated peptide/protein antibodies has had a large impact on routine serological testing [5,6]. Besides being highly specific diagnostic markers for RA, anti-citrullinated peptide/protein antibody tests serve as predictors of disease course and outcome [7-11]. The most widely used and most extensively evaluated anti-citrullinated peptide/protein antibody assay is that developed by van Venrooij and colleagues; that is, IgG-class antibodies to anti-cyclic citrullinated peptides (IgG anti-CCP) [6]. The second-generation anti-CCP2 antibody tests have a diagnostic sensitivity for RA equal to that of agglutinating rheumatoid factor (RF) and a disease specificity of 90% to 99% [5,6]. Like RF, the presence of circulating IgG anti-CCP has been shown to precede clinical onset of disease by several years [9], indicating a pathogenetic role. The combination of HLA-DRB1 genes encoding the shared epitope (SE) and cigarette smoking leads to a markedly increased risk for anti-CCP-positive RA, implying a gene–environment interaction [12-15].

Compared with RF, anti-CCP is a better prognostic marker of an aggressive disease course with radiological progression [7,8,10,11]. Despite the genetic connection, it seems that only anti-CCP – not SE-carriage by itself – is associated with the increased risk of radiological progression [11]. RF is known to occur among all immunoglobulin isotypes. IgA-RF has been stated of particular interest as a predictor of aggressive disease, at least when rabbit IgG is used as the source of antigen for RF detection [16,17]. High circulating levels of RF and immune complexes, in particular IgA-RF and IgA-containing immune complexes, have also been shown to be associated with systemic rheumatoid vasculitis [18,19], and high levels of IgA-RF have been reported to be associated with poor response to TNF inhibitors [20]. The aim of the present study is to analyse to what extent IgA-class antibodies to anti-cyclic citrullinated peptides (IgA anti-CCP) occur in recent-onset RA and how they compare with IgG anti-CCP as a predictor of the disease course.

## Patients and methods

### Patients

Three hundred and twenty patients with recent-onset RA (onset of joint swelling <12 months prior to inclusion) were enrolled in the Swedish TIRA project (Swedish Early Intervention in Rheumatoid Arthritis) during 27 months (1996 to 1998) [8]. Of these patients, 97% fulfilled the 1987 revised American College of Rheumatology classification criteria [21], and the remainder (n = 9) met the following criteria: morning stiffness ≥ 60 minutes, symmetrical arthritis, and arthritis of hands (wrists, metacarpophalangeal or proximal interphalangeal joints) or feet (metatarsophalangeal joints). Serum samples were available from 228 patients at the time of diagnosis, and from 72 of these patients at the 3-year follow-up.

The patients were prescribed disease-modifying anti-rheumatic drugs (DMARDs) as judged appropriate by the treating physicians, who were unaware of both the patients' IgG anti-CCP status and IgA anti-CCP status. The prescription of traditional DMARDs (methotrexate, sulfasalazine, anti-malarial drugs, gold, azthioprine) and/or TNF inhibitors was registered at baseline and after 3, 6, 12, 24, and 36 months. On the same occasions, serum C reactive protein, the 28-joint count Disease Activity Score [22] and the physician's global assessment of disease activity were registered. Functional disability was also assessed at baseline and after 12, 24 and 36 months using the Swedish version of the Health Assessment Questionnaire [23].

The cigarette smoking status (current smoker (smoker at least until 1 year before inclusion), previous smoker, or never smoker) was registered at inclusion as described previously [24]. The SE was assessed as previously described [25]. DNA was available from 171 of the patients.

### Autoantibody analyses

The second-generation IgG anti-CCP (RA immunoscan mark 2; EuroDiagnostica, Arnhem, the Netherlands) were analysed as described previously [9], and a modification of this diagnostic kit was used to analyse anti-CCP antibodies of the IgA class. Patient sera were diluted 1:100 using the diluent provided with the kit. As a secondary antibody we used a horseradish peroxidase-conjugated polyclonal rabbit anti-human α-chain antibody (DakoCytomation, Glostrup, Denmark), which was diluted 1:2,000 with the kit diluent. A seven-step serial dilution of a high-levelled IgA anti-CCP patient serum served as the calibrator and the results were expressed as arbitrary units (AU/ml).

Serum samples were analysed in duplicate and the cutoff limit was set at 25 AU/ml based upon the 99th percentile of 80 blood donors (no differences were seen comparing female and male blood donors). The intra-assay coefficient of variation of the IgA anti-CCP assay was 13% based upon six sera analysed 13 times each, and the inter-assay coefficient of variation (nine separate analyses) was 15%.

Particle agglutinating RF tests, which formed the basis for classification as seropositive RA and seronegative RA, respectively, were performed at the diagnostic laboratories affiliated to the local hospitals participating in the study. Isotype-specific RF analyses (IgM and IgA) were carried out by enzyme immunoassays as described previously [8].

### Statistical analysis

Statistical analyses were performed using SPSS statistical software (version 15.0; SPSS, Chicago, IL, USA). Spearman's rho correlation coefficient was used to detect an association between IgG anti-CCP and IgA-anti-CCP-levels. The Mann–Whitney U test was used to evaluate the difference in IgG-anti-CCP levels between IgA-positive sera and IgA-negative sera, and to assess differences in antibody occurrence/antibody levels between groups with different smoking and SE status. Differences regarding disease activity measures and the Health Assessment Questionnaire at baseline and over time were tested by analysis of variance for repeated measurements. Changes in antibody levels over time were analysed by Wilcoxon's signed-rank test.

### Ethical considerations

The participating patients gave their written informed consent, and the study protocol was approved by the regional ethics committee in Linköping, Sweden.

## Results

Patient characteristics in relation to their IgA anti-CCP status are presented in Table 1.

**Table 1:** Characteristics of the 228 rheumatoid arthritis patients

	Total	IgA anti-CCP-positive, IgG anti-CCP-positive	IgA anti-CCP-negative, IgG anti-CCP-positive
Number of patients	228	66	72
Mean age (years)	55	57	52
Number of women:men	158:70	48:18	46:26
Mean age (years)	54:59	55:62	51:53
Number of agglutinating rheumatoid factor-positive	142	56	68
(%)	63	85	84
IgM-rheumatoid factor-positive (%)	74	98	96
Median level (U/ml)	114	247	184
IgA-rheumatoid factor-positive (%)	73	97	87
Median level (U/ml)	45	83	58
IgG anti-CCP-positive (%)	64	100	100
Median level among positive cases (U/ml)	520	1017	222

IgA anti-CCP, IgA-class antibodies against cyclic citrullinated peptides; IgG anti-CCP, IgG-class antibodies against cyclic citrullinated peptides

### IgA anti-CCP versus IgG anti-CCP results

**Figure 1:**
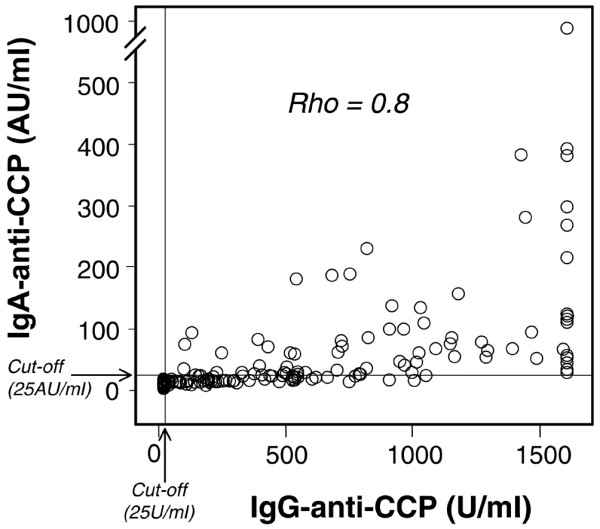
**Correlation of IgA-class and IgG-class antibodies against cyclic citrullinated peptides**. Correlation between IgG-class antibodies against cyclic citrullinated peptides (IgG anti-CCP) levels and IgA-class antibodies against cyclic citrullinated peptides (IgA anti-CCP) levels in 215 early rheumatoid arthritis sera analysed at inclusion.

**Figure 2:**
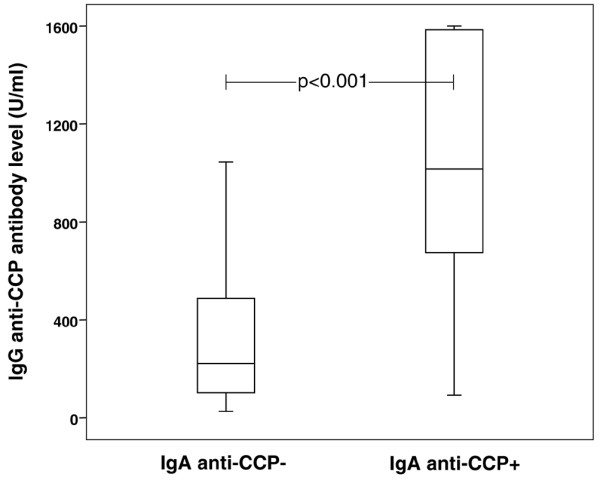
**IgG-class antibody levels (median/75-percentiles) in IgG-positive patients with and without IgA-class antibodies against cyclic citrullinated peptides**. Box plots illustrating the median IgG-class antibodies against cyclic citrullinated peptides (IgG anti-CCP) levels as well as the 75th percentiles and total ranges among the IgG-positive patients with positive IgA-class antibodies against cyclic citrullinated peptides (IgA anti-CCP) tests.

Sixty-six of the 228 inclusion sera (29%) tested positive for IgA anti-CCP, compared with 64% regarding IgG anti-CCP. All sera testing positive for IgA anti-CCP were also positive in the IgG assay, but only 47% of the sera testing positive for IgG anti-CCP had anti-CCP antibodies of the IgA class. As illustrated in Figure 1, the levels of IgG anti-CCP and IgA anti-CCP showed a high degree of correlation (Spearman's rho = 0.8, *P *< 0.01). The IgA-positive sera had significantly higher levels of IgG anti-CCP (*P *< 0.001) as compared with the IgG anti-CCP-positive sera without concomitant IgA anti-CCP (Figure 2).

The status of IgA anti-CCP positivity remained essentially stable at the 3-year follow-up: 93% of patients were therefore unchanged regarding IgA anti-CCP-positivity; one out of the 72 patients available for comparison between inclusion and the 3-year follow-up (1.4%) had changed from negative to positive, whereas four out of 72 patients (5.5%) had changed from positive to negative. The baseline frequency of IgA anti-CCP in these 72 sera did not differ significantly from the remaining 156 patients (31% and 28%, respectively). Regarding changes in the antibody level, eight of the IgA anti-CCP-positive patients showed an increase >30% and seven patients showed a decrease >30%. Overall, however, the median level of IgA anti-CCP in the whole patient material increased from 60 AU/ml to 81 AU/ml (*P *= 0.005).

### IgA anti-CCP status versus disease course

**Figure 3:**
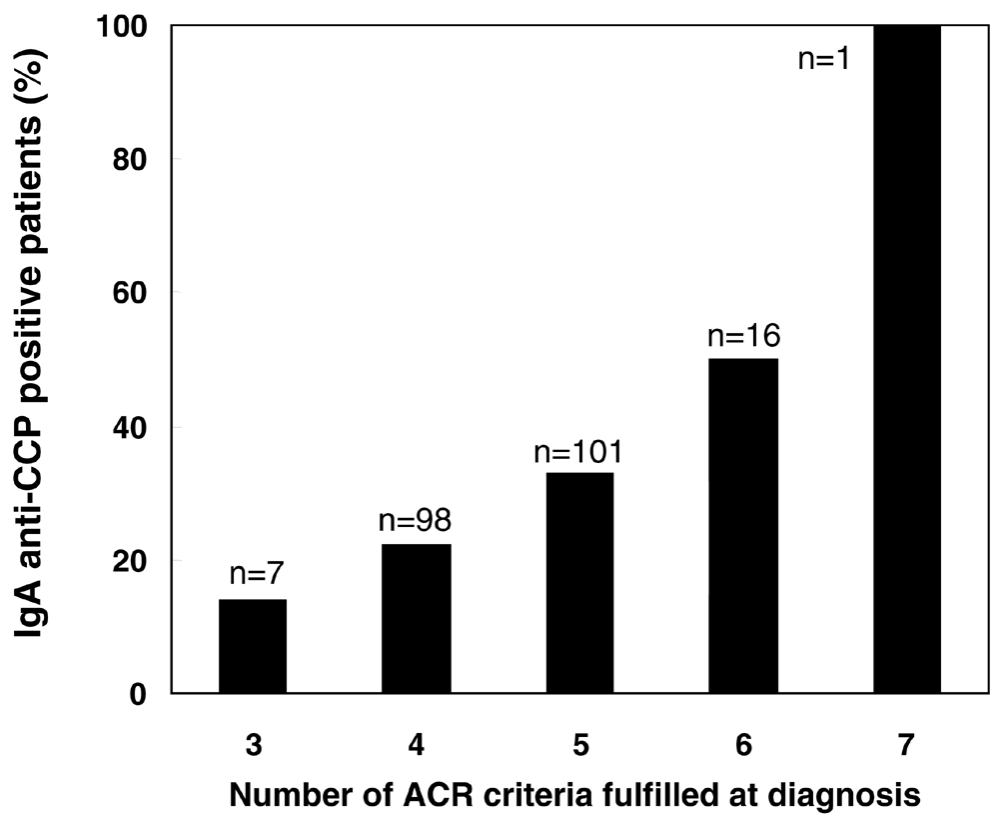
**IgA-class antibody status in relation to baseline number of fulfilled American College of Rheumatology criteria**. Proportion of IgA-class antibodies against cyclic citrullinated peptides (IgA anti-CCP)-positive patients in relation to the number of American College of Rheumatology (ACR) criteria fulfilled at inclusion.

Analysis of the IgA anti-CCP status at baseline in relation to the number of fulfilled American College of Rheumatology classification criteria at inclusion revealed that those patients testing positive had a significantly higher median American College of Rheumatology criterion count (4.73 versus 4.47, *P *= 0.01). As shown in Figure 3, the proportion of IgA anti-CCP-positive cases increased with an increasing count of fulfilled American College of Rheumatology classification criteria.

**Figure 4:**
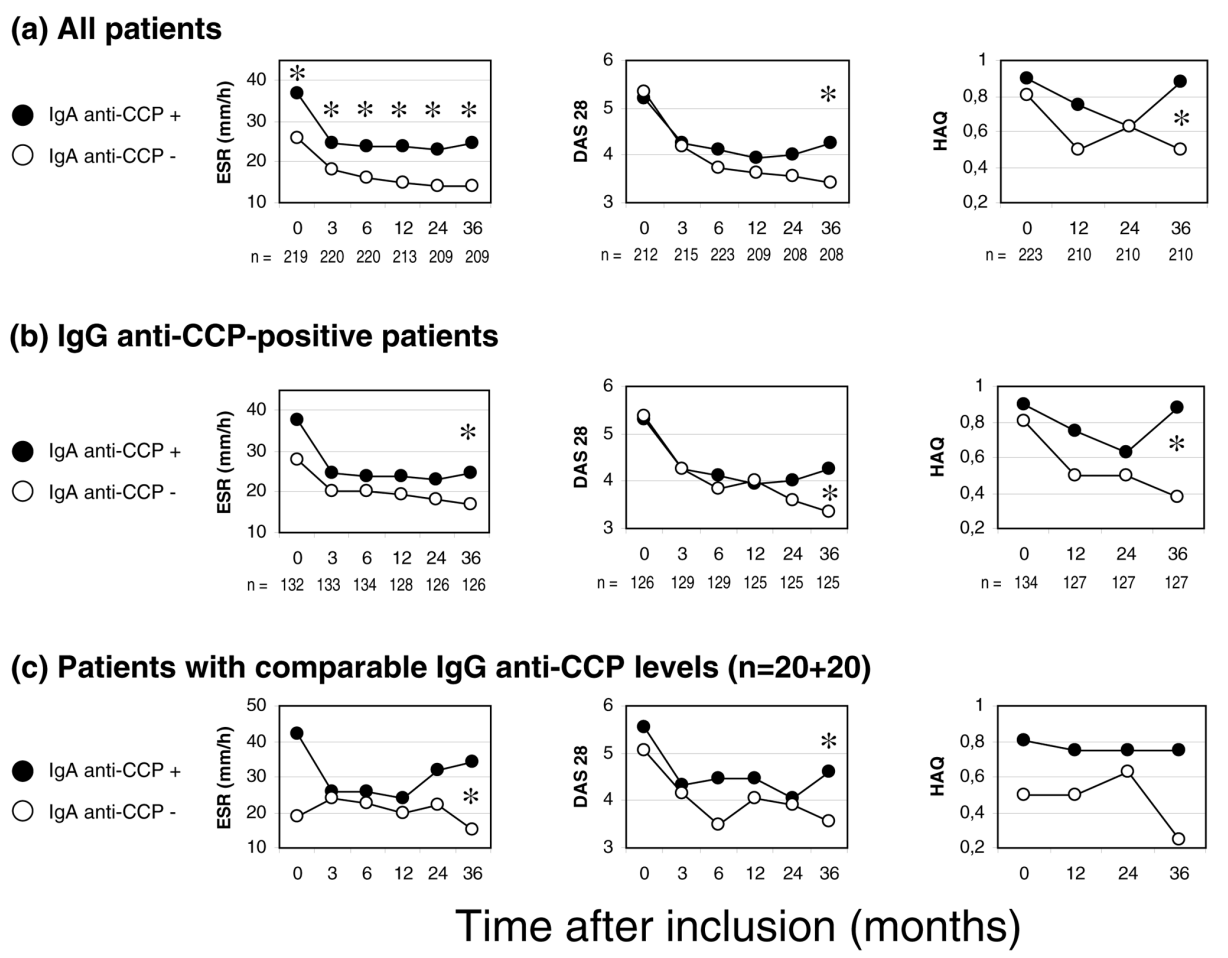
**Disease activity and functional ability measures**. Course of the erythrocyte sedimentation rate (ESR), the 28-joint count Disease Activity Score (DAS28) and the Health Assessment Questionnaire (HAQ) over 3 years. **(a) **All patients. **(b) **IgG-class antibodies against cyclic citrullinated peptides (IgG anti-CCP)-positive RA patients. **(c) **20 IgA-class antibodies against cyclic citrullinated peptides (IgA anti-CCP)-positive patients and 20 IgA anti-CCP-negative patients, with pairwise comparable IgG levels. *P < 0.05.

The erythrocyte sedimentation rate, the 28-joint count Disease Activity Score, and the Health Assessment Questionnaire scores were consistently higher in patients with IgA anti-CCP throughout the 3-year follow-up compared with those patients testing negative (Figure 4a); by analysis of variance for repeated measurements, this increase was statistically significant for the erythrocyte sedimentation rate (*P *= 0.002) and the 28-joint count Disease Activity Score (*P *= 0.0497). This trend remained when comparing cases positive for IgG anti-CCP alone with IgA anti-CCP-positive cases (Figure 4b), but did not reach statistical significance by analysis of variance.

Since the IgA-positive patients had higher average levels of IgG anti-CCP (Figure 2), we made an attempt to evaluate the possible influence of a high IgG anti-CCP level regarding disease progression in IgA anti-CCP-positive patients. Forty patients, 20 IgA-positive and 20 IgA-negative, with pairwise similar (± 15%) IgG anti-CCP levels were identified (range of IgG anti-CCP, 80 to 1,045 U/ml; mean level, 501 U/ml). Comparison of these pairwise IgG-matched IgA-positive/IgA-negative cases revealed the same tendency; that is, IgA anti-CCP-positive patients had a more aggressive disease course over 3 years – although the differences did not reach statistical significance (Figure 4c).

**Figure 5:**
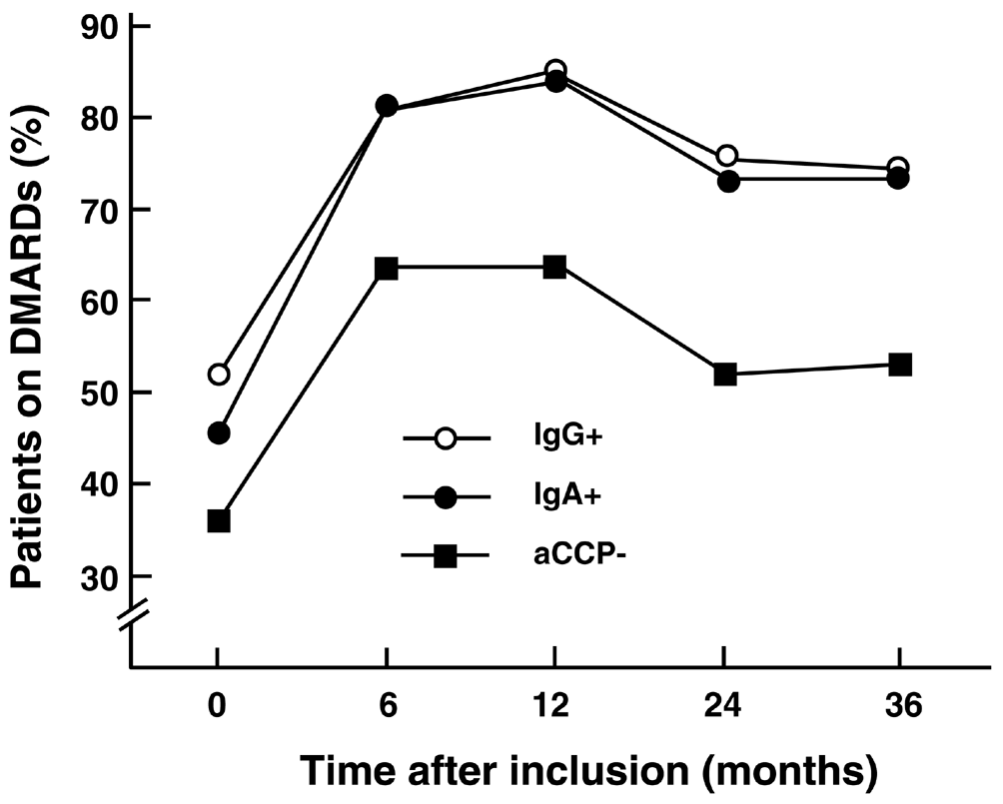
**Prescription of disease-modifying anti-rheumatic drugs in patients with different anti-cyclic citrullinated peptide status**. Diagram to illustrate the prescription of disease-modifying anti-rheumatic drugs (DMARDs) at different time points after enrolment of patients with different anti-cyclic citrullinated peptide (aCCP) status. Prescribing physicians were unaware of the aCCP results.

The higher preparedness to prescribe DMARDs to IgG anti-CCP-positive patients as compared with anti-CCP-negative cases in the present cohort [8] was not further enhanced in the IgA anti-CCP-positive cases (Figure 5). We had no access to radiographic follow-up, but at baseline the presence of typical X-ray findings in hands and feet were comparable between anti-CCP-positive and anti-CCP-negative patients (14% versus 12%) for both IgA-class and IgG-class antibodies.

### IgA anti-CCP status and IgG anti-CCP status versus smoking and shared epitope

Forty-three per cent of the current smokers were IgA anti-CCP-positive (n = 40), compared with 37% among previous smokers (n = 38) and 25% among never smokers (n = 150). The difference between current smokers and never smokers was significant regarding the proportion of patients testing positive for IgA anti-CCP (*P *= 0.027), but not regarding the mean antibody levels of IgA anti-CCP-positive patients. The corresponding difference regarding smoking and IgG anti-CCP status did not reach statistical significance.

When subgrouping the patients based on SE status (double, single, or no SE copies), the presence of IgG anti-CCP strongly correlated to SE occurrence in a dose-dependent manner. In the group with two SE copies, 95% were IgG anti-CCP-positive – compared with 70% in the group with one copy and 33% in the group without a SE. A much weaker (nonsignificant) trend was recorded regarding IgA anti-CCP status versus SE status (39% in SE^+/+^, 27% in SE^+/-^, and 21% in SE^-/-^).

## Discussion

Analysis of IgA-class autoantibodies is well established regarding a few disease states, most notably antibodies against endomysium and tissue transglutaminase in coeliac disease [26,27]. Anti-neutrophil cytoplasmic antibodies of the IgA class have been reported to occur in ulcerative colitis, autoimmune liver diseases, Henoch–Shönlein's purpura, and neutrophilic dermatoses [28-31], and RF of the IgA class has been claimed to be of clinical interest in RA [16-20].

In a study by Verpoort and colleagues in Leiden, IgA anti-CCP antibodies were analysed in early arthritis patients testing positive for IgG anti-CCP [32]. In the present study on early RA we found anti-CCP antibodies of the IgA isotype in 29% of the patients in total, and in 47% of the patients testing positive for anti-CCP antibodies of the IgG class. The reason for the slightly lower proportion of IgA anti-CCP-positive patients in our study compared with the Leiden group may be the tough cutoff limit applied in the present study. The finding that a positive test in the IgA assay was restricted to patients with IgG anti-CCP antibodies, however, confirms the findings of Verpoort and colleagues [32]. In a study by Anzilotti and colleagues, antibodies of the IgA class reacting with a citrullinated viral peptide were identified in a few cases of RA who did not have detectable IgG anti-citrullinated viral peptide antibodies [33].

We and other workers have shown that the presence of IgG anti-CCP is related to a more severe disease course with poorer outcome compared with patients testing negative for IgG anti-CCP [7,8,10]. Similarly, in the present study, patients testing positive for IgA anti-CCP had significantly higher disease activity over 3 years of prospective follow-up compared with those patients testing negative. This may, at least in part, be explained by the concomitant presence of IgG anti-CCP antibodies. The tendency to higher disease activity in the IgA anti-CCP-positive/IgG anti-CCP-positive patients as compared with those with IgG anti-CCP alone could also hypothetically have been related to the higher baseline levels of IgG anti-CCP, since previous studies indicate that high levels of IgG anti-CCP indicate a more severe disease [34]. We found, however, that IgA-anti-CCP-positive patients tended to have a more severe disease course over 3 years as compared with IgA-negative cases with the same baseline levels of IgG anti-CCP. This observation, suggesting that IgA anti-CCP may possibly have an even better predictive potential than IgG anti-CCP regarding disease course, calls for further prospective studies, including radiographic evaluation, in larger patient materials. Similar to the IgG anti-CCP status at baseline and 3-year follow-up [8], the presence or absence of IgA anti-CCP at baseline usually remained after 3 years. Contrary to IgG-class antibody levels in serum, however, the average IgA anti-CCP level had not decreased 3 years after diagnosis and instituted DMARD therapy – but rather, on the contrary, had increased. We think this observation is interesting, but it needs further scrutiny in future studies before any far-reaching conclusions can be made.

Klareskog and colleagues have proposed the hypothesis that cigarette smoking acts as a local airway trigger of excessive citrullination leading to immunisation in SE-positive individuals [12]. SE carriage is not, however, an absolute prerequisite to develop immunity to citrullinated antigens. In the present study, 95% and 70% of the patients with double SE copies and single SE copies, respectively, were IgG anti-CCP-positive, but as many as 33% were positive in the group without SE. The corresponding figures regarding IgA anti-CCP antibodies were 39%, 27% and 21%. Despite the low association with SE, the occurrence of IgA-anti CCP significantly correlated to smoking, which again corroborates recent findings by Verpoort and colleagues [35]. We think the IgA response to citrullinated antigens attracts special interest with regard to mucosal triggers, and we propose that IgA anti-CCP of mucosal origin should be analysed in future studies.

## Conclusion

IgA anti-CCP analysis does not appear to add a diagnostic benefit to IgG anti-CCP analysis alone, but the presence of IgA-class antibodies may predict a more severe disease course in early RA. Further studies are justified to shed more light on this question.

## Abbreviations

CCP = cyclic citrullinated peptides; DMARD = disease-modifying anti-rheumatic drug; HLA = human leukocyte antigen; IgA anti-CCP = IgA-class antibodies against cyclic citrullinated peptides; IgG anti-CCP = IgG-class antibodies against cyclic citrullinated peptides; RA = rheumatoid arthritis; RF = rheumatoid factor; SE = shared epitope; TIRA = Early Intervention in Rheumatoid Arthritis; TNF = tumour necrosis factor.

## Competing interests

The authors declare that they have no competing interests.

## Authors' contributions

AS, AK and TS participated in the conception and design, acquisition of data, analysis and interpretation of the data, and writing the manuscript. ÅR-O participated in acquisition of the data.
